# Quality of Care in One Italian Nursing Home Measured by ACOVE Process Indicators

**DOI:** 10.1371/journal.pone.0093064

**Published:** 2014-03-27

**Authors:** Claudia Pileggi, Benedetto Manuti, Rosa Costantino, Aida Bianco, Carmelo G. A. Nobile, Maria Pavia

**Affiliations:** Department of Health Sciences, University of Catanzaro “Magna Græcia”, Catanzaro, Italy; Cardiff University, United States of America

## Abstract

**Objectives:**

To adapt the Assessing Care of Vulnerable Elders Quality Indicators (ACOVE QIs) for use in Italy, to assess the adherence to these indicators as reported in the medical records of residents in a nursing home (NH), to compare this adherence for general medical and geriatric conditions, and eventually, to identify the relationships between patients' characteristics and reported processes of care.

**Methods:**

Two physicians collected the data by reviewing medical records of all NH residents in the previous 5 years, for a period of one year. Patients aged <65 years were excluded. A total of 245 patients were reviewed during the study period. The ACOVE QIs set, developed for NH processes of care, was used to assess the quality of care. Multivariate analysis was performed to identify and to assess the role of patients' characteristics on quality of processes of care by several domains of care in general medical and geriatric conditions.

**Results:**

With the exception of diabetes management, quality of processes of care for general medical conditions approached adequate adherence. Care falls substantially short of acceptable levels for geriatric conditions (pressure ulcers, falls, dementia). On the contrary, the recommended interventions for urinary incontinence were commonly performed. Adherence to indicators varied for the different domains of care and was proven worse for the screening and prevention indicators both for geriatric and general medical conditions. Statistical analysis showed disparities in provision of appropriate processes of care associated with gender, age, co-morbidities, level of function and mobility, length of stay and modality of discharge by NHs.

**Conclusions:**

Adherence to recommended processes of care delivered in NH is inadequate. Substantial work lies ahead for the improvement of care. Efforts should focus particularly on management of geriatric conditions and on preventive healthcare.

## Introduction

In recent decades an exponential growth in numbers of vulnerable elders has led to the concept of emerging new needs associated with their increasing disabilities, and concomitantly an increasing but transformative new demand for health care requiring formal long-term support. Long-term care represents a program of health care, personal care and social services delivered over time to persons who have lost or never acquired some nominal degree of functional capacity [1]. These services may be provided in a variety of settings, largely accounted for in Nursing Homes (NHs).

Older patients, as highlighted in a recent review article, do not receive appropriate care [2], so innovative quality improvement strategies still need to be designed, developed and implemented in settings now delivering suboptimal care [3], [4]. To explore where, when and for which conditions quality deficiencies exist, Rand Corporation developed a comprehensive set of indicators for assessing the quality of the processes of care, rather than of the outcomes, namely the Assessing Care Of Vulnerable Elders (ACOVE) quality indicators (QIs) [5], [6]. These QIs represent minimum care rather than optimal care to be provided for general medical and geriatric conditions to the vulnerable elders, and are meant to assess and ultimately improve the quality of care [5], [6].

In Italy there is limited experience about the use of standardized indicators to assess the quality of care provided to NH resident elders, and no study has examined quality of processes of care delivered to prevent, diagnose and treat the main general medical conditions compared to the geriatric ones.

The aims of our study were to adapt the ACOVE QIs in a specific geographic area of Italy, to assess the adherence to these indicators as reported in medical records of residents in a NH, to compare this adherence for general medical and geriatric conditions, and eventually, to identify the relationships between patients' characteristics and reported processes of care. Our data may contribute to implement QIs on a large scale, thus promoting the adoption of best practices in NHs.

## Materials and Methods

The study was undertaken in one 40-bed NH facility located in the area of Catanzaro (Italy). The catchment area of Catanzaro encompasses about 240.000 inhabitants and 1.635 sq km. It is served by six NHs for a total of 194 beds.

Two previously trained physicians, who were not involved in care, collected the data by reviewing medical records of NH residents. A detailed protocol has been used to train reviewers to extract data from medical records. In the protocol, simulations of the most common situations that the reviewers would find in the medical records were also included. Finally, the first 30 medical records were reviewed together by two physicians and all discrepancies were resolved through discussion, re-reading and the possible intervention of a third reviewer.

Data were collected for all patients who had been NH residents for at least one year in the previous 5 years (2001 to 2006). If patients were admitted for more than one year, the previous 12 months of stay were evaluated; patients aged less than 65 were excluded. The following data were collected for each patient: socio-demographics, mode of admission to NH, cognitive and functional health status, and delivered processes of care. To assess the clinical conditions of patients, we collected the individual diagnoses and the Cumulative Illness Rating Scale for Geriatrics (CIRS-G index) [7], [8], a clinician-rated scale of cumulative medical burden in geriatric patients. Two CIRS-G indices were used: the 14 CIRS-G severity index, represented by the disease severity mean (ranging from 1, no problem, to 5, extremely severe) in each of 14 organ systems, and the 14 CIRS-G comorbidity index, computed by counting the number of items for which a score of 3 or more was reported. Cognitive status was assessed using the Short Portable Mental Status Questionnaire (SPMSQ) [9], and the level of function and mobility using the Barthel index [10].

The ACOVE QI set, developed for NH processes of care, and covering 11 general medical conditions [11] and 6 geriatric syndromes [12] was used to assess the quality of care.

The ACOVE QIs have been subjected to a cross-cultural translation and adaptation process into the Italian language. The process of cross-cultural adaptation involved several steps: 1) translation from English to Italian; 2) establishment of an expert committee that included two experienced researchers in internal medicine and geriatrics, three researchers proficient in survey design and cross-validation method, one language professional and one translator; 3) meeting of the expert committee to produce the first Italian draft; 4) pilot-testing on a focused group of charts; 5) meeting of the expert committee to produce a new consensus version; 6) back-translation to English; 7) re-evaluation by the committee members and production of the final draft. Then, a pilot study was conducted in order to test the final draft of the tool. Subsequently, the translated version of the tool was submitted to a group of experts who were senior researchers in public health, in internal medicine and geriatrics. They reviewed the format and content of the items, as well as the content validity of the tool as a whole. Eventual disagreement between physicians in reviewing medical records was resolved by discussion.

We selected 4 medical conditions (hypertension, diabetes mellitus, heart failure, pneumonia and influenza) (Appendix S1) and 4 geriatric syndromes (dementia, urinary incontinence, falls and mobility disorders and pressure ulcers) (Appendix S2); the clinical conditions were selected since they were the most frequently encountered ones in the study population. Several indicators were assessed for each condition (Hypertension = 13, Diabetes mellitus = 11, Heart failure = 9, Pneumonia and Influenza = 8, Dementia = 13, Urinary incontinence = 6, Falls and mobility disorders = 6, Pressure ulcer = 7).

The ACOVE QIs are constructed in an IF/THEN format. The "IF" portion of the QI defines the eligible patient for a specific process of care, and the "THEN" portion defines the recommended care. So, "IF" in the patient's medical record that specific clinical characteristic was reported (eg. that NH resident had diabetes), "THEN" it was necessary to check whether the procedure described by the QI had been performed or not (eg. his or her glycosylated hemoglobin levels had been measured at least every 12 month). Therefore, each NH resident has been considered eligible in relation to one or more clinical conditions reported in the medical record. Whenever the condition described by one of the QI appeared in the medical record, a score of 1 was assigned if the process of care had been performed in adherence to the indicator, otherwise a score of 0 was attributed. For each patient the same indicator could be measured several times according to the recurrence of the condition in the medical record. If the patient had an identified contraindication to a process of care, the related indicator was not included in the scoring process. If feasibility of any indicator was questionable, it was not considered [12], [13]. For each clinical condition, scores were calculated at the patient level as the percentage of adherence to the recommended process of care. For example, a patient who had 1 medical conditions (hypertension), and 1 geriatric syndrome (dementia), might have been eligible for all 13 hypertension QIs, and for 11 of the 13 dementia QIs. If 7, and 4 QIs, respectively, were satisfied, the patient's mean quality score for hypertension would be calculated as 7∶13 = 54%, and for dementia as 4∶11 = 36%. Moreover, the scores were also calculated by domain of care, categorized into three groups: screening and prevention, diagnosis and treatment. For instance, of the 13 indicators related to hypertension 3 belonged to the screening and prevention domain of care, 4 to the diagnosis, and 6 to the therapy. If the 7 QIs satisfied were divided as the following: 1 QI in screening and prevention, 2 QIs in diagnosis and 4 QIs of therapy, the patient's mean quality score for each domain of care related to hypertension would be calculated as 1∶3 = 33% for screening and prevention, as 2∶4 = 50% for diagnosis, and as 4∶6 = 67% for therapy.

Therefore we provided one QI score for each eligible clinical condition and for each domain of care.

The Ethics Committee of the "Mater Domini" Hospital of Catanzaro (Italy) approved the protocol of the study. As a matter of course, written consent was always requested when admission to the NH occurred, and only the patients who had given permission for their personal data to be used for research were included in the study.

### Statistical analysis

In the primary analysis, we used t-test to compare the mean level of the QIs scores for general medical and geriatric conditions in the three domains of care. Six multiple linear regression models were developed to evaluate the relationship of baseline independent characteristics with: QIs scores calculated in the three domains of care (screening and prevention, diagnosis and therapy), separated for general medical and geriatric conditions.

In all 6 models the explanatory variables included were the following: age (≤75 years = 1, 76–85 years = 2, ≥86 years = 3), sex (male = 0, female = 1), marital status (single/divorced/widowed = 0, married = 1), education level (no formal education = 0, ≥5 years = 1), referral to NH (hospital/residential care services = 0, home = 1), length of NH stay (≤18 months = 0, 19–36 months = 1, ≥37 months = 2), outcome of NH stay (categorical, discharge = 1, death = 2, transfer to another facility = 3, NH attendance = 4), 14 CIRS-G comorbidity index (continuous), 14 CIRS-G severity index (continuous), Barthel index (total independence = 0, dependence: mild = 1, moderate = 2, severe = 3, total = 4), SPMSQ (cognitive function impairment: no = 0, borderline or mild = 1, moderate = 2, severe = 3), medical/surgical examinations (no = 0, yes = 1), referral to emergency department (no = 0, yes = 1), hospital admission (no = 0, yes = 1). Regression coefficients (β), standard errors (SE) and 95% confidence intervals (CI) were calculated. All of the tests for significance were two-sided and p-values ≤0.05 were considered statistically significant. All analyses were conducted using the Stata software program, version 11 [14].

## Results

Medical records for 245 patients were reviewed during the study period, and their main characteristics are presented in Table 1. More than half were referred to NH by hospitals or residential care services (56.3%), and the mean length of stay was 27.3 months (standard deviation +15.8). Percentages of eligible patients varied for each clinical condition, ranging from 26.5 for heart failure to 100 for pneumonia and influenza. Adherence to quality processes of care according to clinical conditions investigated is found in Appendix S1 and Appendix S2.

**Table 1:** Selected Characteristics of the Study Population.

Characteristic	N	%	Mean+SD^a^
Sex			
Male	105	42.9	
Female	140	57.1	
Age group, years			81.7+8.2
≤75	55	22.4	
76–85	94	38.4	
≥86	96	39.2	
Educational level, years of schooling			
None	132	53.9	
5	77	31.4	
8	21	8.6	
≥13	15	6.1	
Marital status			
Single/Divorced/Widowed	205	83.7	
Married	40	16.3	
Access to NH^b^ care			
Hospital/Residential care services	138	56.3	
Home	107	43.7	
Length of stay in the NH^b^, months			27.3+15.8
≤18	101	41.2	
19–36	86	35.1	
≥36	58	23.7	
Outcome of NH^b^ stay			
Discharge	81	33.1	
Death	63	25.7	
Transferred to another facility	39	15.9	
Still in NH^b^	62	25.3	
Access to the Emergency Department			
None	176	71.8	
≥1	69	28.2	
Medical/Surgical investigation received			
No	33	13.5	
Yes	212	86.5	
Cognitive function impairment (SPMSQ^c^)			
Intact mental functioning	3	1.2	
Borderline or mild impairment	21	8.6	
Moderate impairment	135	55.1	
Severe impairment	86	35.1	
Level of function and mobility (Barthel index)			
Totally independent	2	0.8	
Mild dependence	19	7.8	
Moderate dependence	52	21.2	
Severe dependence	85	34.7	
Total dependence	87	35.5	
14 CIRS-G^d^ Comorbidity Index			5.9+2.1
14 CIRS-G^d^ Severity index			2.1+0.4

a Standard Deviation; ^b^Nursing Home; ^c^ hort Portable Mental Status Questionnaire; ^d^Cumulative Illness Rating Scale For Geriatrics.

A detailed examination of the appropriateness of care for individual conditions revealed that, among general medical conditions (Appendix S1), the mean level QI score for recommended processes of care for hypertension was 77.6%; almost all subjects had regular follow-up checks and received appropriate pharmacological management, but orthostatic blood pressure was rarely checked. The mean level QI score for diabetes mellitus was 67.3%; almost all diabetics received regular blood pressure checks, annual foot examination and glycosylated haemoglobin checks, but fewer than 25% received an ophthalmologic examination. The mean level QI score for heart failure management was 81.8%; high compliance to specific physical examinations and complete medical history was found, but appropriate beta blockers prescriptions were provided only to 31% of those eligible. Mean adherence rates for pneumonia and influenza, even though all patients were eligible, was 75.1%; almost all recommended interventions showed high compliance for patients with pneumonia. Formal strategies to increase vaccinations were usually adopted (85%), but influenza vaccine was administered three times more often than pneumococcal vaccine (90% vs 29%).

Concerning geriatric conditions (Appendix S2), the mean level QI score for dementia reached 57%. In more than 90% of residents with cognitive impairment a validated cognitive assessment was performed, but only 34% were screened for depression. No medical records were found to indicate the registration of any bracelet identification. The mean level QI score for recommended interventions to prevent falls and treat mobility disorders was 72.7%. Of eligible subjects, 96% were examined for balance or gait disturbances at admission, and more than two-thirds enrolled in exercise programs; however, hypotension screening was documented in only 14%. The mean level QI score for pressure ulcers was 63.2%. Appropriate risk assessment at admission and at recommended intervals was performed in 40% of eligible subjects, whereas 84% of residents with a pressure ulcer received this type of evaluation. For more than 90% of subjects appropriate topical therapy was applied, but adequate nutritional assessment was performed in only 22%. Compliance with urinary incontinence indicators was 82.7%. Among recommended interventions for urinary incontinence, the risk assessment at admission, the appropriateness of indications and documentation for catheterization and chronic indwelling catheter use were frequently followed and performed. In contrast, appropriate toileting programs and behavioral treatments were less frequently applied (25%).

The adherence to indicators varied for different domains of care and was worse for screening and prevention for both geriatric (7.3%) and general medical conditions (26.1%), compared to diagnosis indicators for geriatric (37.5%) and general medical conditions (49.5%). As shown in Table 2, globally, a lower adherence to recommended processes of care was registered for geriatric compared to general medical conditions. Indeed, univariate analysis results showed a significant negative relationship of adherence in geriatric compared to general medical conditions in diagnostic (69.3%±32.6% vs 82.2%±20.7%%; p<0.001), therapeutic (48.4%±37.3% vs 67.6%±32.3%; p<0.001) and screening and prevention (43.6%; ±28.7% vs 72.1%±24.5%; p<0.001) domains of care.

**Table 2:** Adherence to quality processes of care for general medical versus geriatric conditions by domain of care.

Type of care	Diagnosis		Treatment		Screening and Prevention	
	Qls^a^ measured	Mean adherence rate+SD^b^	Qls^a^ measured	Mean adherence rate+SD^b^	Qls^a^ measured	Mean adherence rate+SD^b^
	N (%)	%	N (%)	%	N (%)	%
**General medical condition**	192 (78.4)	82.2+20.7	219 (89.4)	67.6+32.3	245 (100)	72.1+24.5
**Geriatric condition**	224 (91.4)	69.3+32.6	174 (71)	48.4+37.3	245 (100)	43.6+28.7
**Mean difference (+SD^b^)**		12.9+37.7		19.3+49.7		28.5+36.1
**Adherence to QIs** a		t = 4.5, 172 df, p<0.001		t = 4.87, 157 df, p<0.001		t = 12.3, 242 df, p<0.001

a Quality Indicators; ^b^Standard Deviation.

Results of the multiple linear regression analysis are presented in Table 3. Adherence to diagnostic recommended processes for general medical conditions was significantly higher in older patients (β-coeff = 10.6, p<0.001), males (β-coeff = −6.2, p = 0.028), married subjects (β-coeff = 8.8, p = 0.013), with lower length of stay at NH (β-coeff = −4.7, p = 0.007), with higher 14 CIRS-G severity index (β-coeff = 18.7, p = 0.004) and lower 14 CIRS-G comorbidity index (β-coeff = −3.1, p = 0.015), and in patients who had not been referred to the emergency department (β-coeff = −7.2, p = 0.018), whereas it was significantly lower in patients discharged from NH (β-coeff = −9.2, p = 0.01) compared to those who died. Finally, a greater level of function and mobility was associated to better adherence to diagnostic QIs for general medical conditions (β-coeff = −3.1, p = 0.051).

**Table 3:** Multivariate linear regression analysis indicating association between several variables and the different outcomes.

	Model 1			Model 2			Model 3			Model 4			Model 5			Model 6		
	Quality of diagnostic process in general medical conditions			Quality of diagnostic process in geriatric conditions			Quality of therapy process in general medical conditions			Quality of therapy process in geriatric conditions			Quality of screening and prevention process in general medical conditions			Quality of screening and prevention process in geriatric conditions		
Variable	Coef	SE	p	Coef	SE	p	Coef	SE	p	Coef	SE	p	Coef	SE	p	Coef	SE	p
Sex	−6.2	2.81	0.028	−8.9	4.50	0.049	*-* a			−8.2	5.74	0.155	−3.7	3.31	0.266	*-* a		
Age group, years	10.6	1.87	<0.001	3.0	2.88	0.299	*-* a			−8.9	3.93	0.025	*-* a			*-* a		
Educational level, years of schooling	*-* a			−7.9	4.52	0.082	−6.1	4.28	0.157	*-* a			4.7	3.47	0.181	*-* a		
Marital status	8.8	3.53	0.013	−6.2	5.82	0.288	*-* a			*-* a			−5.2	4.25	0.220	*-* a		
Access to NH^b^ care	*-* a			*-* a			*-* a			*-* a			−3.3	3.13	0.297	*-* a		
Length of stay in the NH, months	−4.7	1.71	0.007	*-* a			4.5	2.68	0.095	*-* a			−2.8	2.03	0.172	−3.3	2.24	0.141
Outcome of NH^b^ stay																		
Death	*Ref.*			*Ref.*			*Ref.*			*Ref.*			*Ref.*			*Ref.*		
Discharge	−9.2	3.56	0.010	9.3	5.77	0.107	−7.7	4.48	0.086	*-* a			*-* a			−8.9	4.04	0.028
Transferred	−8.2	4.47	0.067	−8.2	6.70	0.223	*-* a			12.1	9.01	0.181	4.8	4.36	0.272	−8.9	5.03	0.079
Still in NH	4.7	3.80	0.218	11.7	5.91	0.049	*-* a			8.0	6.52	0.224	9.7	3.80	0.011	*-* a		
Access to the ED^c^	−7.2	3.01	0.019	*-* a			5.7	4.46	0.207	−5.5	6.23	0.375	*-* a			−4.5	4.01	0.264
Hospital admission	−3.5	2.95	0.242	*-* a			*-* a			*-* a			*-* a			−4.9	3.88	0.208
Examinations received	*-* a			*-* a			−7.7	6.23	0.219	*-* a			*-* a			*-* a		
Cognitive function impairment ^d^	*-* a			*-* a			*-* a			*-* a			−3.5	2.53	0.161	*-* a		
Level of function and mobility^e^	−3.1	1.58	0.051	*-* a			−4.9	2.32	0.035	4.1	3.07	0.180	*-* a			3.4	1.99	0.092
14 CIRS-G^f^ Comorbidity Index	−3.1	1.26	0.015	*-* a			5.3	1.08	<0.001	*-* a			*-* a			*-* a		
14 CIRS-G^f^ Severity index	18.7	6.35	0.004	21.0	5.38	<0.001	*-* a			13.6	7.82	0.083	*-* a			14.7	4.80	0.002

a Variable removed by *Backward Elimination*; ^b^Nursing Home; ^c^Emergency Department; ^d^Short Portable Mental Status Questionnaire; ^e^Barthel index; ^f^Cumulative Illness Rating Scale For Geriatrics.

Adherence to diagnostic recommended processes in geriatric conditions was significantly associated with higher 14 CIRS-G severity index (β-coeff = 21, p<0.001), male gender (β-coeff = −8.9, p = 0.049) and with being still resident in NH (β-coeff = 11.7, p = 0.049).

Adherence to therapy recommended processes for general medical conditions was significantly associated with higher 14 CIRS-G comorbidity index (β-coeff = 5.3, p<0.001) and better level of function and mobility (β-coeff = −4.9, p = 0.035), whereas for geriatric conditions, it was significantly higher in younger subjects (β-coeff = −8.9, p = 0.025).

Finally, in the regression models performed to investigate the adherence to screening and prevention recommended processes for general medical conditions it was significantly better in patients still resident in NH (β-coeff = 9.7, p = 0.011), whereas for geriatric conditions it was significantly better in patients with higher 14 CIRS-G severity index (β-coeff = 14.7, p = 0.002) and worse in patients discharged from NH (β-coeff = −8.9, p = 0.028).

## Discussion

Unlike acute hospital care and primary care activity, the quality of long-term care for the elderly provided in NHs has long been neglected. To our knowledge, this is the first study performed in Italy that has evaluated the quality of care delivered in this setting by using a validated set of indicators that reflect the adherence to current evidence-based processes of care.

The results of our study show that appropriateness of processes of care for vulnerable elders is extremely variable according to ACOVE QIs, both in different conditions and in specific domains of care. Among general medical conditions, diabetes management showed the greatest deficiencies. This result is congruent with other studies reporting poor quality of care in NHs for diabetes, particularly in respect to preventive interventions [15], [16], and may reflect serious difficulties in the access to specialized services [17]. In contrast, adequate adherence to recommended processes of care was found for other general conditions, such as heart failure, hypertension and pneumonia. One explanation for this disparity may be inherent in the skills necessary for many processes of care related to these conditions [13].

Significant deficiencies exist in most geriatric conditions, such as pressure ulcers, falls and dementia, while recommended interventions for urinary incontinence were commonly performed. This higher adherence for urinary incontinence management may be attributed to administrative and organizational factors in our healthcare system since safeguards against incontinence (pads/diapers and/or catheters) are free of charge for incontinent patients. On the contrary, in accordance with previous studies [18], [19], appropriate toileting programs and behavioral treatments are adequately performed in only 25% of the eligible patients; one possible explanation for this disparity may be detectable in the attitudes of physicians who often overlook the potential efficacy of these behavioral interventions [19].

Our findings, consistent with studies showing poor implementation of evidence-based processes of care for geriatric as compared to general medical conditions [20], suggest the need for improving the training of healthcare professionals in these specific health needs. Certain interventions, such as the management of physical restraints and the use of identification bracelets, are not incorporated as protective standard healthcare processes in our context.

It is worrisome that low adherence to recommended processes of care is particularly pronounced for geriatric conditions in the screening and prevention domains of care, and this lack of oversight may be due to negative attitudes of health professionals in NHs to the usefulness of preventive care in the elders. Indeed, counseling or screening may be perceived as insufficient and inefficient time-consuming activities, whereas treatment or diagnostic domains, often involving simple processes of care, such as prescription of medications or ordering diagnostic tests, are considered more effective, less bothersome, and tasks easier to perform [13]. These results are quite consistent with those in a previous study conducted by some of us to estimate the adherence to evidence-based processes of care in acute settings, that highlighted the need to focus effort for improvement initiatives especially in the area of preventive care [21].

Unlike most previously published studies conducted with aggregated data, our findings derived from a smaller number of patients; nonetheless detailed information was gathered from each patient and represents a main strength of our study allowing us to point to factors that could predict adherence to ACOVE QIs, such as the clinical conditions, the socio-demographics of patients and features of long term care, for example, admission, discharge and length of stay in NH. Indeed, the results from the multivariate analyses confirm the many disparities argued in previous studies [22]–[24], women appear to receive less appropriate interventions for diagnosis of general and geriatric conditions, and older patients are more likely to receive adequate diagnostic processes of general medical conditions. Consistent with previous studies that examined the effect of coexisting conditions on providing appropriate processes of care [22]–[24], interestingly, we found that quality of care, as measured by the ACOVE QIs, is not worse in more complex patients. An increased 14 CIRS-G severity index of comorbidity was significantly associated with more adequate prevention of geriatric conditions and diagnosis of general and geriatric conditions.

Some potential limitations of the present study must be acknowledged. Processes of care were determined using medical record documentation which might be incomplete; it can be argued that availability and the quality of data correlates with lower estimates of adherence rates, and as underlined in previous surveys [13], poorer documentation is likely to be correlated with poorer processes of care. Data in our study were collected in a single NH, and concern relating to generalizability and comparability of the results may arise. The present study is intended to be an analytical first step in measuring the adherence to current evidence-based processes of care reported in medical records of residents in NHs in an area of Italy by using a specialized set of indicators. The application of ACOVE QIs, in our experience, provides valuable information in relation to their feasibility and ease of use, suggesting that these indicators, once tested in a wider context, might be implemented on a large scale for the evaluation of the quality of processes of care in NHs. On the other hand, it is known that there are significant differences between Northern and Southern Italy for many health services indicators, and despite the scarce data available in Italy about healthcare in NHs, it has already been reported that these settings appear to be inadequate to the healthcare needs of vulnerable elders [25], [26]. Direct comparisons to the few studies conducted in Italy on quality of care in NHs is problematic since the goals for quality assessment were different. For example, Donini el al. [27] assessed the perceived quality in food and nutritional care in a NH in Rome (Central Italy); Garavaglia et al. [28] investigated quality of care in Northern Italian NHs mainly in terms of costs, and Moro et al. [29] described the prevalence of infections in NH residents as critical components of the quality of care in long-term facilities. Despite these differences, the findings from these studies are consistent with ours, and they all highlight the need of a more in depth evaluation of care provided to NH residents. Although we cannot dismiss the supposition that our results pertain only to our limited area, our findings strongly suggest the opportunity to expand the assessment of quality of care in NHs through the use of ACOVE QIs in Italy.

## Conclusions

Our findings reveal significant deficiencies in the adherence to recommended processes of care delivered in NH and suggest that there is still substantial work that lies ahead on the road to improvement of care. Our study both calls attention to these deficiencies and confirms the usefulness of ACOVE QIs to measure and compare performance. Efforts in the future should focus particularly on management of geriatric conditions and on the specific domain of preventive healthcare provided to elders.

## Supporting Information

Appendix S1 **Adherence to ACOVE process indicators for the management of general medical conditions in NH residents.** NH =  nursing home; D =  diagnosis; T =  therapy; SP =  screening and prevention; ACE =  angiotensin-converting enzyme; HF =  heart failure; HTN =  hypertension.
(DOC)

Appendix S2 **Adherence to ACOVE process indicators for the management of geriatrics syndromes in NH residents.** NH =  nursing home; D =  diagnosis; T =  therapy; SP =  screening and prevention; PCP =  primary care practitioner; MDS =  minimum data set; UI =  urinary incontinence.
(DOC)
