# Supplementary material for: Quality of Care in One Italian Nursing Home Measured by ACOVE Process Indicators
Source: PLoS One. 2014 Mar 27;9(3):e93064. doi: 10.1371/journal.pone.0093064 (PMC3968054; doi:10.1371/journal.pone.0093064)
Supplement: Appendix S1 — Adherence to ACOVE process indicators for the management of general medical conditions in NH residents. NH = nursing home; D = diagnosis; T = therapy; SP = screening and prevention; ACE = angiotensin-converting enzyme; HF = heart failure; HTN = hypertension. (DOC) [file pone.0093064.s001.doc]

**Appendix S1. Adherence to ACOVE process indicators for the management of general medical conditions in NH** residents

| **Topic** | **Domain of care** | **NH indicator** | **N. eligible patients (%)** | **Mean aderence rate (+SD)** | **% eligible patients who met the indicator** |
| --- | --- | --- | --- | --- | --- |
| **Hypertension**  **(13 indicators)** |  |  | **169 (69)** | **77.6 (+15.7)** |  |
| Hypertension diagnosis | D | IF a NH resident’s blood pressure (BP) is elevated  THEN at least two follow-up BP reading should be obtained in the next month if systolic BP>160 mm Hg or diastolic BP>100 mm Hg or within month if systolic BP=150-160 mm Hg or diastolic BP=90-100 mm Hg. | 131 (53.5) |  | 95.4 |
| Physical examination | D | IF a NH resident is diagnosed with new hypertension  THEN a physical examination within 4 weeks of the diagnosis should include a fundoscopic eye examination, a lung examination, a cardiac examination (including evaluation of pulses), an abdominal examination (including assessment for bruits) and an extremity examination. | 9 (3.7) |  | 77.8 |
| Electrocardiogram for new hypertension | D | IF a NH resident is newly diagnosed with hypertension  THEN an electrocardiogram and blood chemistries (sodium, potassium, creatinine and fasting glucose) should be performed within 4 weeks of the diagnosis. | 9 (3.7) |  | 77.8 |
| Cardiovascular risk documentation | D | IF a NH resident is newly diagnosed with hypertension  THEN there should be documentation regarding the presence or absence of other cardiovascular risk factors. | 8 (3.3) |  | 100 |
| Hypertension intervention | T | IF a vulnerable elder remains hypertensive  THEN he or she should be offered a therapeutic intervention to lower BP within 3 months if systolic BP 161-180 mm Hg or within 1 month if systolic BP >180mm Hg. | 14 (5.7) |  | 71.4 |
| Pharmacologic management | T | IF a NH resident remains hypertensive after nonpharmacologic intervention  THEN pharmacologic antihypertensive treatment should be initiated. | 10 (4.1) |  | 90 |
| Measure orthostatic blood pressure | T | IF a NH resident is treated with antihypertensive medication  THEN both supine and standing BP should be measured with each adjustment of BP medication | 156 (63.7) |  | 19.9 |
| Follow-up HTN treatment | SP | IF a NH resident is diagnosed with hypertension and pharmacologic intervention is initiated  THEN follow-up blood pressure checks should occur every 2 weeks until BP control<150/90 mm Hg or targeted BP goal has been achieved. | 8 (3.3) |  | 100 |
| Follow-up controlled HTN | SP | IF a NH resident with hypertension is treated with pharmacologic therapy and has achieved BP control (<150/90 mm Hg) or targeted BP goal  THEN follow-up BP checks should occur at least every 3 months | 145 (59.2) |  | 100 |
| Follow-up lytes for diuretic | SP | IF a NH resident is prescribed a diuretic  THEN he or she should have serum electrolytes (including blood urea nitrogen or creatinine) checked within 7 days after initiation of therapy, after dose adjustment, and at least yearly. | 120 (49) |  | 83.3 |
| Malignany HTN | T | IF a NH resident develops a hypertensive emergency with a diastolic BP>120mm Hg and with manifestations of critical target organ damage (and no do not hospitalize order exists)  THEN parenteral hypertensive therapy to reduce mean arterial BP by 25%acutely and diastolic BP to 100-110 mm Hg within the nest several hours should be initiated while the patient is in a monitored setting in the hospital. | 29 (11.8) |  | 89.7 |
| Pharmacologic management renal disease | T | If a NH resident has hypertension and renal parenchymal disease with a serum creatinine concentration greater than 1.5 mg/dl or more then 1 g of protein/24 hours of collected urine  THEN therapy whit an ACE inhibitor should be offered. | 8 (3.3) |  | 75 |
| Pharmacologic management beta-blocker therapy | T | IF a NH resident has hypertension should not be used  THEN beta-blocker therapy for hypertension should not be used | 78 (31.8) |  | 97.4 |
| **Diabetes mellitus**  **(11 indicators)** |  |  | **90 (36.7)** | **67.3 (+18.9)** |  |
| Monitor glycated hemoglobin (D) | D | IF a NH resident has diabetes  THEN his or her glycosylated hemoglobin levels should be measured at least every 12 month or care goals or other records should indicate why this is non appropriate. | 90 (36.7) |  | 60.7 |
| Improve glycemic control | T | IF a NH resident has an elevated glycosylated haemoglobin level  THEN he or she should be offered a therapeutic intervention aimed at improving glycemic control within 1 month for Hgb A1C >9.0% or care goals or other records should indicate why this is not appropriate. | 14 (5.7) |  | 85.7 |
| Examine feet | D | IF a NH resident has diabetes  THEN his or her feet should be examined by the primary care provider at least annually. | 90 (36.7) |  | 63.3 |
| Measure BP | D | IF a NH resident has diabetes  THEN his or her BP should be checked monthly. | 90 (36.7) |  | 100 |
| Intervene for glu>300 | T | IF a NH resident has a glucose level of >300 mg/dL  THEN specific therapeutic intervention aimed at glycemic control should be initiated within 2 weeks or care goals or other records should indicated why this is not appropriate. | 26 (10.6) |  | 92.9 |
| Change diet if losing weight | T | IF a NH resident with diabetes mellitus and body weight <120% of ideal, has adequate glycemic control Hgb A1C<9.0 or glucose<200 and is losing weight on a special diet  THEN the resident should be changed to a regular diet. | 5 (2) |  | 20 |
| Examine eyes | D | IF a NG resident with diabetes is not blind and does not have existing retinopathy  THEN he or she should receive a dilated eye examination performed by a specialist in diabetes, an ophthalmologist or an optometrist at least every 2 years. | 80 (32.7) |  | 23.8 |
| Treat proteinuria | T | IF NH resident with diabetes has proteinuria  THEN he or she should be offered therapy with ACE inhibitor or ACE receptor blocker. | 10 (4.1) |  | 50 |
| Aspirin therapy | T | ALL NH residents with diabetes, who are not on other anticoagulant therapy, should be offered daily aspirin therapy. | 70 (25.6) |  | 68.6 |
| BP control | T | IF a NH resident with diabetes has elevated BP with BP>160/100 mm Hg  THEN he or she should be offered a therapeutic intervention to lower BP within 3 months. | 26 (10.6) |  | 96.2 |
| Treatment of high cardiovascular risk | T | IF a diabetic vulnerable elder has one additional cardiac risk factor (ie, smoker, hypertension, hypercholesterolemia, or renal insufficiency/microalbuminuria)  THEN he or she should be offered an ACE inhibitor or receptor blocker | 67 (27.3) |  | 56.7 |
| **Heart failure (HF)**  **(9 indicators)** |  |  | **65 (26.5)** | **81.8 (+20.3)** |  |
| Evaluate ejection fraction in new HF | D | IF a NH resident is newly diagnosed with heart failure  THEN he or she should be offered an evaluation of his or her left ventricular ejection fraction within 1 month. | 21 (85.7) |  | 61.9 |
| Indication for diuretics | T | IF diuretics are given to a NH resident  THEN the indication for the diuretic should be stated in the medical record. | 48 (19.6) |  | 93.8 |
| Medical history in setting of HF | D | IF a NH resident has a diagnosis of heart failure  THEN a history should be taken at the time of NH admission and/or new diagnosis that documents the presence or absence of the following: current symptoms of chest pain or angina, documented coronary artery disease, revascularization, hypertension, diabetes, hypercholesterolemia, valvular heart disease, thyroid disease, alcohol use, smoking, current medications, and functional capacity (eg, New York Heart Association functional status). | 61 (24.9) |  | 82 |
| Physical examination | D | IF a NH resident is diagnosed with heart failure  THEN, at the time of NH admission or new diagnosis, the following elements of the physical examination should be documented: weight, BP, heart rate, results of lung, cardiac and abdominal or lower-extremity examination. | 65 (26.5) |  | 98.5 |
| Diagnostic testing for HF | D | IF a NH resident is newly diagnosed with HF  THEN the following studies should be done within 1 month of diagnosis (unless the tests were performed within the prior 3 months): chest radiography; electrocardiograph; complete blood count; measurement of serum sodium, potassium, and creatinine; thyroid-stimulating hormone (TSH) in resident with atrial fibrillation or HF with no obvious etiology. | 27 (11) |  | 70.4 |
| Follow-up after HF hospitalization | SP | IF a NH resident returns to the NH after hospitalisation for HF  THEN he or she should have follow up that includes weight measurement within 7 days after hospital discharge. | 5 (2) |  | 0 |
| ACE inhibitor use | T | IF a NH resident has HF and left ventricular ejection fraction of 40% or less  THEN he or she should be offered an ACE inhibitor or an angiotensin receptor antagonist. | 25 (17.2) |  | 92 |
| Use beta blocker in HF | T | IF a NH resident has HF, has left ventricular election fraction of 40% or less, and New York Association class I to III disease  THEN he or she should be offered a beta blocker unless a contraindication (for example, uncompensated HF) has been indicated. | 13 (5.3) |  | 30.8 |
| Monitor digoxin | T | If a NH resident with HF is treated with digoxin  THEN the digoxin level should be checked within 1 week if additional medications are added than could affect digoxin level (quindine, verapamil, amiodarone) or if signs of toxicity develop. | 39 (15.9) |  | 61.5 |
| **Pneumonia and influenza (8 indicators)** |  |  | **245 (100)** | **75.1 (+22.2)** |  |
| Antibiotics in NH | T | If a NH resident is diagnosed with pneumonia  THEN antibiotics should be administered within 8 hours of diagnosis. | 72 (29.4) |  | 97.2 |
| Oxygen therapy in NH | T | IF a NH resident treated for a NH-acquired pneumonia has hypoxia  THEN the resident should be transferred to a hospital or receive oxygen therapy in the NH or the record should document why that is not indicated. | 57 (23.3) |  | 96.5 |
| Hospitalized pneumonia | T | IF a NH resident with pneumonia has unstable vital signs, despite a trial of NH based therapy and does not have a do not hospitalize order  THEN the resident should be transferred to the hospital or the record should document why that is not indicated or document advanced dementia or poor prognosis. | 18 (7.3) |  | 88.9 |
| Pneumococcal vaccination | S | IF a NH resident with no history of allergy to the pneumococcal vaccine is not know to have received a pneumococcal vaccine or received the vaccine more than 5 years ago (if before age 65)  THEN a pneumococcal vaccine should be offered. | 245 (100) |  | 28.6 |
| Influenza vaccination | SP | IF a NH resident has no history of anaphylactic hypersensitivity to eggs or to the other components of the influenza vaccine  THEN the resident should be offered an annual influenza vaccination. | 245 (100) |  | 92.2 |
| Vaccination rates | SP | IF pneumococcal and/or influenza vaccination rates among residents of a NH are low (less than 90% of institutionalized elderly)  THEN methods to increase the rate of vaccination should be used. | 244 (99.6) |  | 85.3 |
| Vaccinate health care workers | SP | ALL NHs should have a formal plan to offer and encourage influenza vaccination among their employees. | 242 (98.8) |  | 83.9 |
| Changing parenteral to oral antibiotics | T | IF a NH resident with NH-acquired pneumonia is to be switched from parenteral to oral antimicrobial therapy  THEN unless intravenous access cannot be maintained, the resident should meet the following criteria: a clinically improving condition (ie, improved cough, resolved fever, decreased leukocytosis); hemodynamic stability (ie, heart rate <100 beats/min; systolic BP > mm Hg ;respiratory rate <24 breaths/min; oxygen saturation >90% on room air); tolerance of oral medication or food and fluids. | 17 (6.9) |  | 94.1 |

NH = nursing home; D = diagnosis; T = therapy; SP = screening and prevention; ACE = angiotensin-converting enzyme; HF = heart failure; HTN = hypertension
