# Supplementary material for: Quality of Care in One Italian Nursing Home Measured by ACOVE Process Indicators
Source: PLoS One. 2014 Mar 27;9(3):e93064. doi: 10.1371/journal.pone.0093064 (PMC3968054; doi:10.1371/journal.pone.0093064)
Supplement: Appendix S2 — Adherence to ACOVE process indicators for the management of geriatrics syndromes in NH residents. NH = nursing home; D = diagnosis; T = therapy; SP = screening and prevention; PCP = primary care practitioner; MDS = minimum data set; UI = urinary incontinence. (DOC) [file pone.0093064.s002.doc]

**Appendix S2. Adherence to ACOVE process indicators for the management of geriatrics syndromes in NH residents**

| **Topic** | **Domain of care** | **NH indicator** | **N. eligible patients (%)** | **Mean aderence rate (+SD)** | **% eligible patients who met the indicator** |
| --- | --- | --- | --- | --- | --- |
| **Dementia**  **(13 indicators)** |  |  | **243 (99.2)** | **57.3 (+21.9)** |  |
| Cognitive screen for new admission | SP | IF a vulnerable elder is admitted to the NH  THEN the physician, other primary care provider, or specialist should document the results of a cognitive assessment with a validated instrument such as the Folstein Mini-Mental Status Exam. | 240 (98) |  | 94.2 |
| Evaluate memory loss | D | IF a NH resident without a previous diagnosis of dementia fails a memory screen or presents with memory loss or forgetfulness  THEN the physician should document an assessment of memory or a diagnosis of treatment for dementia (or cognitive dysfunction or forgetfulness) or an explanation for the memory loss or a referral to neurology, psychiatry, geriatrics or psychology. | 15 (6.1) |  | 66.7 |
| Re-evaluate corrected impairment | D | IF a NH resident without delirium or a potentially reversible cognitive impairment has the problem corrected  THEN the physician should document that he or she has reviewed either the next minimum data set (MDS) cognitive score or has performed another cognitive evaluation within 6 month. | 132 (53.9) |  | 61.4 |
| Caregiver support and patient safety | SP | IF a NH resident has cognitive impairment  THEN on admission, a healthcare provider should obtain a history about resident safety (including wandering and other problematic behaviours), observe resident behaviour, and establish a behavioural management plan than includes how staff will deal with conflicts in the NH. | 229 (93.5) |  | 23.1 |
| ID bracelet | SP | IF a NH resident who is demented is at risk for wandering  THEN the resident should wear identification. | 65 (26.5) |  | 0 |
| Document consent and targets for restraints | SP | IF a NH resident is to be physically restrained  THEN the target behavioural disturbance or safety issue justifying use of the restraints must be identified to the consenting person (resident or legal guardian) and documented in the chart. | 48 (19.6) |  | 8.3 |
| Identify restraint alternatives | SP | IF a NH resident is placed in physical restraints  THEN potential management strategies other than physical restraints should be documented by the healthcare team. | 28 (11.4) |  | 39.3 |
| Review medications | T | IF a NH resident has new or worsening cognitive impairment  THEN the physician should review the resident’s medication list for initiation of medications that might correspond chronologically to the onset of dementia symptoms. | 32 (13.1) |  | 53.1 |
| Change medication | T | IF a NH resident presents with new or worsening cognitive impairment that corresponds in time with the star of new medication(s)  THEN the physician should discontinue or justify the necessity of continuing these medications. | 16 (6.5) |  | 93.8 |
| Screen for depression | SP | IF a NH resident has dementia  THEN he or she should be screened for depression during the initial evaluation period. | 166 (67.8) |  | 34.3 |
| Treat depression | T | IF a NH resident with dementia has depression  THEN he or she should be treated for the depression. | 107 (43.7) |  | 45.8 |
| Laboratory testing | D | IF a NH resident is newly diagnosed with dementia  THEN serum levels of vitamin B12 and thyroid-stimulating hormone (TSH) should be measured. | 6 (2.4) |  | 16.7 |
| Stroke prophylaxis | SP | IF a NH resident with mild to moderate dementia has cerebrovascular disease  THEN the resident should be offered appropriate stroke prophylaxis. | 89 (36.3) |  | 48.3 |
| **Falls and mobility disorders**  **(6 indicators)** |  |  | **243 (99.2)** | **72.7 (+29.2)** |  |
| Examine balance and gait | SP | ALL NH residents should have a note documenting a physicians or primary care provider (PCP) examination for the presence of balance or gait disturbances on admission. | 242 (98.8) |  | 95.9 |
| Evaluate frequent falls | D | IF a NH resident has had two or more falls in a month  THEN in the 30 days before or after the second fall, the physician or PCP should either perform a basic fall evaluation or document that this represents an ongoing problem that has been evaluated. | 21 (8.6) |  | 57.1 |
| Evaluate gait/mobility and balance | D | IF a NH resident reports or the MDS documents new or worsening difficulty with ambulation, balance, or mobility  THEN there should be physician documentation that a basic gait, mobility, and balance evaluation was performed within 2 months that resulted in specific diagnostic impressions and therapeutic recommendations. | 64 (26.1) |  | 65.6 |
| Offer exercise for strengthening | SP | IF a NH resident who is capable of exercise is found to have problems with gait, strength (eg, four out or five or less on manual muscle testing), endurance(eg, dyspnea on mild exertion), or needs to use his or her arms to rise from a chair  THEN an exercise program should be offered. | 49 (20) |  | 69.4 |
| Screen for Hypotension | SP | IF a NH resident is taking a medication that commonly causes hypotension  THEN the PCP should document postural changes in BP and pulse at least once. | 127 (51.8) |  | 14.2 |
| Evaluate postural hypotension | D | IF a NH residents found to have postural hypotension  THEN the physician note should document further evaluation for possible causative factors (eg, diabetes, medications). | 78 (31.8) |  | 66.7 |
| **Pressure ulcers**  **(7 indicators)** |  |  | **93 (38)** | **63.2 (+24.1)** |  |
| Assess risk | SP | IF a NH resident is unable to reposition himself or herself, or has limited ability to do so  THEN a risk assessment using a multidimensional standardized scale (eg, the Braden scale or Norton scale) should be performed on admission and every week during the first 4 weeks. | 93 (38) |  | 39.8 |
| Initiate prevention | SP | IF a NH resident is identified as “at risk” for pressure ulcer development or a pressure ulcer risk assessment score indicates that the person is “at risk”  THEN within 24 hours preventive intervention must address all of the following : 1) repositioning by written schedule every 2 hours or there should be documentation that repositioning is not needed or not tolerated; 2) pressure reduction (or management of tissue loads) unless there is documentation that this is not needed or not tolerated; and 3) nutritional status. | 82 (33.5) |  | 24.4 |
| Evaluate pressure ulcer | D | IF a NH resident is found to have a pressure ulcer  THEN the pressure ulcer should ne assessed for lo location, depth and stage, size and presence of necrotic tissue. | 69 (28.2) |  | 84.1 |
| Assess nutrition | T | IF a NH resident is found to have a pressure ulcer  THEN a nutritional assessment should me preformed within 1 week by a dietician or a PCP. | 67 (27.3) |  | 22.4 |
| Debride necrotic tissue | T | IF a NH resident presents with a full-thickness sacral or trochanteric pressure ulcer covered with necrotic debris or eschar  THEN debridement by using sharp, mechanical, enzymatic, or autolytic procedures should be done within 3 days of diagnosis. | 31 (12.7) |  | 64.5 |
| Do not use topical antiseptic | T | IF a NH resident has a stage or greater pressure ulcer  THEN a topical antiseptic should not be used on the wound. | 42 (17.1) |  | 92.9 |
| Topical dressing | T | IF a NH resident presents with a clean full-thickness or a partial-thickness pressure ulcer  THEN a moist wound-healing environment should be provided with topical dressing. | 50 (20.4) |  | 98 |
| **Urinary incontinence (UI)**  **(6 indicators)** |  |  | **241 (98.4)** | **82.7 (+24)** |  |
| Screen for UI | SP | ALL NH residents should have documentation of the presence or absence of UI at the time of admission. | 241 (98.4) |  | 98.3 |
| Obtain history | D | IF a NH resident has UI on admission or the new onset of UI that persist for over 1 month  THEN a targeted history should be obtained that documents each of the following: 1) characteristics of voiding, 2) ability to get to the toilet, 3) prior treatment for UI, 4) importance of the problem to the patient, and 5) mental status. | 141 (55.6) |  | 48.9 |
| Document catheterization reasons | T | IF NH resident has a chronic urinary retention and overflow UI, us not candidate for a more definitive procedure, does not have severe physical or mental impairments and indwelling urethral catheterization is used  THEN there should be documentation in the medical or NH record that he or she has 1) terminal illness or 2) has pressure ulcers in the relevant area, or 3) that resident prefers indwelling catheter to an intermittent or suprapubic catheter. | 9 (3.7) |  | 88.9 |
| Chronic indwelling catheter use | T | IF a NH resident has clinically significant overflow UI, and indwelling urethral catheterization is used  THEN there should be documentation that the resident is not a candidate for alternative interventions ad a result of severe physical or mental impairments or does not want alternative interventions. | 28 (11.4) |  | 75 |
| Offer behavioural therapy | T | IF a cognitively intact NH resident who is capable of independent toileting has documented stress, urge, or mixed incontinence without evidence of hematuria or high post void residual  THEN behavioural treatment should be offered. | 53 (21.6) |  | 24.5 |
| Consider behavioural or pharmacologic therapy | T | IF NH resident with a post-void residual <200 cc continues to have two or more incontinence episodes/day despite receiving assisted toileting two times/die  THEN the resident should be offered either behavioural or pharmacologic therapy in combination with the assisted toileting program. | 24 (9.8) |  | 20.8 |

NH = nursing home; D = diagnosis; T = therapy; SP = screening and prevention; PCP = primary care practitioner; MDS = minimum data set; UI = urinary incontinence
